# China’s top 10 achievements in hematology in 2023

**DOI:** 10.1097/BS9.0000000000000195

**Published:** 2024-06-07

**Authors:** Shuang Liu, Biao Chen

**Affiliations:** a Chinese Journal of Hematology, National Clinical Research Center for Blood Diseases, Haihe Laboratory of Cell Ecosystem, Institute of Hematology & Blood Diseases Hospital, Chinese Academy of Medical Sciences & Peking Union Medical College, Tianjin 300020, China; b Tianjin Institutes of Health Science, Tianjin 300020, China; c Union Hospital, Tongji Medical College, Huazhong University of Science and Technology, Wuhan 430022, China

At the 4^th^ Annual Meeting of the Chinese Alliance for Societies of Hematology (CASH) on January 7, 2024, the “China’s Top 10 achievements in hematology in 2023” were unveiled. This prestigious selection, initiated by the CASH since 2020, has been held successfully for the past three consecutive years.^1^ It aims to document, showcase, and promote significant scientific progress and landmark achievements in the field of hematology research in China. This initiative demonstrates the strength and academic level of China’s hematology research, inspires physicians and scientists in the field to further embrace their missions and strive for excellence, and fosters public understanding, concern, and support for hematology research, creating a positive scientific atmosphere across society.

The scientific papers recommended for the “China’s Top 10 achievements in hematology in 2023” were all published between January 1 and December 31, 2023, with the core scientific discoveries made in China. In 2023, a total of 3505 hematology-related articles were published by Chinese researchers in the Web of Science, marking an increase from 3002 articles in 2022. This upsurge in publication volume underscores the dynamic growth and scholarly activity within China’s hematology research community.

The organizing committee of the Annual Meeting of the CASH invited nominations from 78 experts across 16 sub-disciplines within the field of hematology. After multiple rounds of organization and widespread expert recommendations, 19 achievements entered a list from which the final 10 were selected. This selection was conducted through an online voting process, which included votes from academicians related to the blood field; the Chinese Society of Hematology, Chinese Medical Association; the Chinese Society for Experimental Hematology; the Chinese Association for Blood Sciences; the Hematology Oncology Committee, Chinese AntiCancer Association; the Chinese Hospital Association Hematology Branch; the Chinese Association of Blood Diseases; and the editorial board of *Chinese Journal of Hematology* and *Blood Science*. A total of 126 valid votes were received, with the 10 most-voted achievements constituting the “China’s Top 10 achievements in hematology in 2023.” The selected advances balance clinical and basic research and are characterized by outstanding originality, distinctive innovation, and significant social impact. The following are abstracts summarizing these top 10 achievements.

## 1. ELUCIDATING THE ORIGINS AND LINEAGE SPECIFICATION OF EARLY HEMATOPOIESIS IN PRIMATES VIA EX VIVO THREE-DIMENSIONAL (3D) CULTURE OF MACAQUE EMBRYOS

A groundbreaking study published in the field of hematology has, for the first time, unveiled the dynamics of early hematopoietic origins and lineage specification in macaques at the single-cell level, utilizing an ex vivo long-term 3D culture system.^2^ This research has meticulously characterized the 2 waves of hematopoiesis occurring in the yolk sac, shedding light on the conserved and divergent aspects of early hematopoietic development between primates (humans and macaques) and rodents (mice). As the first international exploration into the origins of early hematopoiesis in macaques, this work has not only demystified the process of hematopoietic lineage specification in the early mesoderm of primates but has also paved the way for cross-species research in hematopoietic development, encompassing human, macaque and murine models.

This study’s innovative approach to understanding the fundamental mechanisms of hematopoiesis in non-human primates offers a novel perspective on the evolutionary biology of blood-cell formation. The findings have significant implications for the development of therapeutic strategies for hematologic disorders and contribute to the broader scientific community’s understanding of the conserved and species-specific aspects of hematopoiesis. The meticulous dissection of the hematopoietic process in macaques provides a valuable foundation for future research, potentially leading to advances in regenerative medicine and the treatment of blood-related diseases.

## 2. TARGETING PROGRAMMED DEATH (PD)-1 TO ERADICATE LEUKEMIA STEM CELLS IN ACUTE T-LYMPHOBLASTIC LEUKEMIA

In a significant breakthrough for the treatment of acute T-lymphoblastic leukemia (T-ALL), a disease with limited therapeutic options and poor prognosis, a recent study has uncovered a novel approach to combat the disease at its source.^3^ The research has, for the first time, demonstrated that PD-1, a cell surface receptor, can specifically identify and label T-ALL leukemia stem cells, which are responsible for disease initiation and drug-resistant relapse. The study has confirmed, in multiple animal models and patient samples, that blocking PD-1 can effectively eliminate leukemia stem cells, leading to a marked therapeutic effect against drug-resistant relapses of T-ALL.

This discovery has profound implications for the precise identification of T-ALL leukemia stem cells and the development of targeted treatment strategies. By focusing on the PD-1 pathway, this research opens new avenues for therapeutic intervention, offering a promising avenue for the management of T-ALL. The findings suggest that PD-1 blockade could be a potent strategy to target and destroy the very cells that drive the persistence and relapse of T-ALL, potentially leading to more effective, personalized treatment options for patients.

Featured as the cover article of the current issue, this study represents a significant step forward in the battle against T-ALL, providing a new understanding of the disease’s pathogenesis and highlighting the potential of immunotherapeutic strategies in the fight against hematologic malignancies. The innovative insights gained from this research could revolutionize the way we approach the treatment of not only T-ALL but also other cancers that rely on similar mechanisms of resistance and persistence.

## 3. PRECISION TREATMENT STRATEGIES FOR DIFFUSE LARGE B-CELL LYMPHOMA BASED ON MOLECULAR SUBTYPING

Two articles from Weili Zhao’s team^4,5^ have introduced a novel approach to the precision treatment of diffuse large B-cell lymphoma (DLBCL) by identifying 20 key pathogenic genes to establish a simplified molecular subtyping. This study has delineated the characteristic signaling pathways for each subtype and confirmed targeted strategies accordingly. In a pioneering move, the research has validated the efficacy and safety of the R-CHOP-X regimen, tailored to molecular subtyping, and has contributed a distinct “Chinese voice” to the global conversation on precision medicine in oncology.

This study represents a significant step toward personalized therapy in DLBCL, aligning treatment with the tumor’s genetic profile for potentially improved outcomes. The findings, presented in 2 influential papers, highlight the potential of targeted therapies and pave the way for future advances in the management of DLBCL and other hematologic malignancies.

## 4. UNRAVELING THE MYSTERY OF ANEMIA AND THROMBOCYTOSIS IN CANCER PATIENTS

Xiaohui Zhang’s research has shed light on the key mechanisms underlying tumor-associated anemia and thrombocytosis, pinpointing abnormal differentiation of megakaryocyte-erythroid progenitors (MEPs) as the critical factor.^6^ The study has elucidated the AHR-RUNX1 signaling pathway as a regulator of abnormal MEP differentiation. Inhibition of this pathway has been shown to reverse the abnormal differentiation of MEPs in cancer patients and in humanized mouse models.

Given that several inhibitors of the activated aryl hydrocarbon receptor (AhR) are currently undergoing clinical trials, this research not only holds significant theoretical importance but also considerable clinical relevance. The findings have the potential to inform new therapeutic strategies for managing anemia and thrombocytosis in cancer patients, offering a promising avenue for improving patient outcomes and quality of life.

## 5. SINGLE-CELL ANALYSIS UNVEILS HEMATOPOIESIS RECONSTITUTION PATTERNS IN ALLOGENEIC HEMATOPOIETIC STEM CELL TRANSPLANT RECIPIENTS

A pioneering study has, for the first time, charted hematopoietic reconstitution landscape in patients undergoing allogeneic hematopoietic stem cell transplantation (allo-HSCT) at the single-cell level.^7^ This research has identified a granulocyte progenitor cell population within hematopoietic stem and progenitor cells (HSPCs) that serves as an indicator for acute graft-versus-host disease (aGVHD), offering invaluable insights into the transplantation ecology of allogeneic HSPCs.

The study’s abstract provides a comprehensive overview of the findings: following allo-HSCT, transplanted HSPCs exhibited rapid and significant changes, particularly a robust proliferative response observed on the first day post-transplantation. Through transcriptomic analysis, the research team discovered an enrichment of immunoregulatory neutrophil progenitors expressing high levels of the S100A gene family in granulocyte colony-stimulating factor-mobilized peripheral blood stem cells. Notably, transplant recipients who later developed aGVHD had a lower infusion of these S100A^high^ immunoregulatory neutrophil progenitors, characterized as Lin^–^CD34^+^CD66b^+^CD177^+^, compared with those who did not.

This groundbreaking research not only enhances our understanding of the regenerative dynamics of transplanted HSPCs in human patients but also identifies a potential biomarker for early risk stratification of patients prone to aGVHD. The identification of this specific progenitor cell population could lead to the development of targeted interventions to mitigate aGVHD, thereby improving the safety and efficacy of allo-HSCT as a treatment for various diseases.

## 6. INTEGRATIVE SINGLE-CELL TRANSCRIPTOMIC ANALYSIS REVEALS HETEROGENEITY OF NATURAL KILLER CELLS ACROSS CANCER TYPES

Recent scientific findings from the team led by Zemin Zhang have shed new light on the heterogeneity of natural killer (NK) cells within the tumor microenvironment in various cancer types.^8^ Through an integrative single-cell RNA sequencing analysis, encompassing data from 716 cancer patients with 24 different cancer types, the study delineated the composition and phenotypic diversity of NK cells in a tumor-specific context.

The research uncovered a distinct subset of tumor-associated NK cells that are not only enriched within tumors but also exhibit diminished anti-tumor capabilities. These cells were found to correlate with a poorer prognosis and increased resistance to immunotherapy, underscoring their clinical significance. Intriguingly, the study also highlighted the potential of myeloid cells, particularly LAMP3^+^ dendritic cells, in modulating NK cell anti-tumor functions, suggesting a complex interplay within the immune response to cancer.

This comprehensive analysis not only advances our understanding of NK cell dynamics in cancer immunity but also identifies specific NK cell subsets that could serve as novel therapeutic targets. The study’s findings are poised to inform the development of biomarkers and targeted treatments, potentially transforming cancer immunotherapy.

## 7. ANTI-G PROTEIN-COUPLED RECEPTOR, CLASS C GROUP 5 MEMBER D (GPRC5D) CHIMERIC ANTIGEN RECEPTOR (CAR)-T CELLS: A PROMISING NOVEL THERAPEUTIC OPTION FOR RELAPSED/REFRACTORY MULTIPLE MYELOMA

Anti-GPRC5D CAR-T cell therapy is showing promise for the treatment of relapsed or refractory multiple myeloma (MM), particularly for patients who have not responded to previous anti-BCMA CAR-T treatments. This innovative approach has demonstrated notable efficacy with an overall high response rate and complete remission in a significant proportion of patients.

The study utilized a phase II trial to administer anti-GPRC5D CAR-T cells following lymphodepletion.^9^ Preliminary outcomes suggest a robust therapeutic potential and a manageable safety profile, marked by common yet mild adverse effects. These developments position anti-GPRC5D CAR-T cells as a potent alternative in the treatment of MM, offering new hope for patients with few remaining treatment options.

## 8. UNVEILING THE HEMATOLOGIC ECOSYSTEM IN OMICRON COVID-19 INFECTIONS

Featured as the cover article in *Immunity*, the research has made a groundbreaking contribution to the field of hematology.^10^ By focusing on the metabolic imbalances within the COVID-19 Omicron variant-infected patients’ blood ecosystems, the study has not only identified unique platelet and immunologic characteristics but also suggested a proactive role for platelets in the pathophysiology of COVID-19.

A standout achievement of this research is the creation of a precision model that employs a multimodal analysis of the blood ecosystem to forecast COVID-19 relapses. This patented innovation marks a significant advancement in the clinical management of the disease.

The introduction of the “blood ecosystem”^11^ concept into scientific discourse highlights the intricate relationship between blood components during Omicron infections. This body of work has expanded our grasp of COVID-19’s hematologic implications and paves the way for future research and therapeutic strategies.

## 9. FIRST INSIGHTS INTO MM PATHOGENESIS AND RESISTANCE VIA METABOLIC DISRUPTIONS

A collaborative body of work from a dedicated research team has brought to light critical metabolic disturbances within the pathology of MM.^12,13^ Through the application of metabolomics, the team has made a significant stride in understanding the disease’s progression and the mechanisms underlying drug resistance.

In the bone marrow microenvironment of MM patients, an imbalance in amino acid metabolism has been observed, with serine metabolism being particularly perturbed. The research indicates that serine, through its involvement in the methionine cycle, impedes the formation of megakaryocytes and contributes to thrombocytopenia, a common complication in MM patients.

Furthermore, the team has elucidated a new mechanism of drug resistance in MM, highlighting the interplay between gut nitrogen-recycling microbes and the host’s metabolism. The study suggests that the accumulation of ammonium ions (NH_4_^+^), facilitated by these microbial interactions, can induce drug resistance in MM.

These discoveries are pivotal, as they not only shed light on the metabolic underpinnings of MM but also propose potential therapeutic targets. By targeting the metabolic pathways that regulate amino acid availability and utilization, the research team has paved the way for novel treatment strategies that could transform the management of MM.

## 10. STING ACTIVATION IN PLATELETS: UNVEILING PATHOGENESIS AND THERAPEUTIC STRATEGIES IN SEPSIS-INDUCED THROMBOSIS

Research has pinpointed the activation of the innate immune molecule STING in platelets as a significant cause of thrombus formation in sepsis, offering new therapeutic prospects for severe infections.^14^ The study is the first to identify the CGAMP/STING/STXBP2 signaling pathway as a critical regulator in platelet activation and thrombogenesis during sepsis.

A proprietary C-ST5 peptide developed by the team has shown potential in treating thrombotic diseases triggered by sepsis, presenting a valuable clinical translation opportunity. This discovery provides a novel targeted strategy for clinical treatment of sepsis-related thrombotic disorders, marking a significant advancement in the field.
